# Validation of patient- and GP-reported core sets of quality indicators for older adults with multimorbidity in primary care: results of the cross-sectional observational MULTIqual validation study

**DOI:** 10.1186/s12916-023-02856-0

**Published:** 2023-04-17

**Authors:** Ingmar Schäfer, Josefine Schulze, Katharina Glassen, Amanda Breckner, Heike Hansen, Anja Rakebrandt, Jessica Berg, Eva Blozik, Joachim Szecsenyi, Dagmar Lühmann, Martin Scherer

**Affiliations:** 1 Department of General Practice and Primary Care, University Medical Center Hamburg-Eppendorf, Martinistr. 52, 20246 Hamburg, Germany; 2 Department of General Practice and Health Services Research, Heidelberg University Hospital, Heidelberg, Germany; 3 Institute of Primary Care, University Hospital Zurich, University of Zurich, Zurich, Switzerland

**Keywords:** Multimorbidity, Quality measurement, Primary care, Comorbidity, Validation study

## Abstract

**Background:**

Older adults with multimorbidity represent a growing segment of the population. Metrics to assess quality, safety and effectiveness of care can support policy makers and healthcare providers in addressing patient needs. However, there is a lack of valid measures of quality of care for this population. In the MULTIqual project, 24 general practitioner (GP)-reported and 14 patient-reported quality indicators for the healthcare of older adults with multimorbidity were developed in Germany in a systematic approach. This study aimed to select, validate and pilot core sets of these indicators.

**Methods:**

In a cross-sectional observational study, we collected data in general practices (*n* = 35) and patients aged 65 years and older with three or more chronic conditions (*n* = 346). One-dimensional core sets for both perspectives were selected by stepwise backward selection based on corrected item-total correlations. We established structural validity, discriminative capacity, feasibility and patient-professional agreement for the selected indicators. Multilevel multivariable linear regression models adjusted for random effects at practice level were calculated to examine construct validity.

**Results:**

Twelve GP-reported and seven patient-reported indicators were selected, with item-total correlations ranging from 0.332 to 0.576. Fulfilment rates ranged from 24.6 to 89.0%. Between 0 and 12.7% of the values were missing. Seventeen indicators had agreement rates between patients and professionals of 24.1% to 75.9% and one had 90.7% positive and 5.1% negative agreement. Patients who were born abroad (− 1.04, 95% CI =  − 2.00/ − 0.08, *p* = 0.033) and had higher health-related quality of life (− 1.37, 95% CI =  − 2.39/ − 0.36, *p* = 0.008), fewer contacts with their GP (0.14, 95% CI = 0.04/0.23, *p* = 0.007) and lower willingness to use their GPs as coordinators of their care (0.13, 95% CI = 0.06/0.20, *p* < 0.001) were more likely to have lower GP-reported healthcare quality scores. Patients who had fewer GP contacts (0.12, 95% CI = 0.04/0.20, *p* = 0.002) and were less willing to use their GP to coordinate their care (0.16, 95% CI = 0.10/0.21, *p* < 0.001) were more likely to have lower patient-reported healthcare quality scores.

**Conclusions:**

The quality indicator core sets are the first brief measurement tools specifically designed to assess quality of care for patients with multimorbidity. The indicators can facilitate implementation of treatment standards and offer viable alternatives to the current practice of combining disease-related metrics with poor applicability to patients with multimorbidity.

**Supplementary Information:**

The online version contains supplementary material available at 10.1186/s12916-023-02856-0.

## Background

Older adults with multimorbidity represent a growing segment of the population [1, 2]. Studies suggest that between 50 and 62% of patients aged 65 years and older are affected by multimorbidity, even if a conservative definition of multimorbidity such as the presence of three or more chronic health conditions is used [3, 4]. Patients with multimorbidity are at greater risk of adverse health outcomes, including poor quality of life and functional limitations, and use more services of the healthcare system [5–8]. They are often faced with complex medication and self-management regimens to manage their multiple health problems [9, 10]. Failure to coordinate care and prioritise treatment goals in line with patient preferences might lead to burdensome and fragmented care [11, 12].

Metrics to assess the quality, safety and effectiveness of care could help policy makers and healthcare providers to respond to the needs of this growing population [13]. However, valid measures for the quality of care for patients with multimorbidity are lacking [14, 15]. Therefore, there is a need to define and operationalise, e.g. through quality indicators, elements of high-quality care for multimorbidity [15]. Quality indicators are metrics referring to structures, outcomes and processes [16, 17]. They are used for quality assurance and monitoring as well as for the empirical evaluation of quality improvement efforts [18]. The call for empirically validated quality indicators specific to multimorbidity is becoming more and more frequent in the scientific literature [13, 15, 19, 20], but validation studies remain scarce [21, 22].

The MULTIqual project aims to develop and validate quality indicators for the primary care of older adults with multimorbidity in Germany. In a multi-step process, 51 candidate indicators had been derived from a systematic literature review and focus groups with patients and their family members. Subsequently, a multidisciplinary expert panel had rated these indicators in the dimensions significance, strength of evidence, possibility to influence the indicator manifestation and clarity of definition. Using nominal group technique, the expert panel then selected a set of 24 quality indicators from general practitioner (GP) perspective and 14 quality indicators from patient perspective and defined a conceptual framework that mapped the indicators to quality dimensions [23, 24].

This study constitutes the final stage of MULTIqual, in which core sets of quality indicators were selected, validated and piloted. The aims of this study were therefore (1) to select patient- and GP-reported core sets of quality indicators that coherently represent the quality dimensions of primary care for patients with multimorbidity; (2) to examine the structural validity, discriminative capacity, feasibility and patient-professional agreement of the selected indicators and (3) to assess the construct validity of the resulting quality scores.

A priori, we expected that all of the quality dimensions identified by expert consensus would be associated with each other and could therefore be represented by one-dimensional core sets of feasible quality indicators. We also hypothesised that the quality scores would be associated with socio-demographic data, health condition, intensity of treatment, patient satisfaction and the patients’ willingness to use GPs as coordinators of their treatment.

## Methods

### Study design and recruitment of participants

We conducted a cross-sectional observational study based on standardised personal interviews with GPs and their patients. Patients were recruited from 35 cooperating GP practices in the cities of Hamburg and Heidelberg and their surrounding areas. The GPs were asked to compile a list of all patients in their practice who met the inclusion criteria. Patients were included if (1) they were aged 65 years or older, and (2) they had at least one consultation in the last completed quarter (i.e. 3-month accounting period) prior to the time of recruitment. From this list, patients were then randomly selected and checked for exclusion criteria until 30 eligible individuals were identified. In seven of the practices, this process was repeated to recruit additional patients.

In our study, multimorbidity was defined by the presence of three or more diseases which are (1) common, (2) chronic, (3) frequently co-occurring with other diseases and (4) potentially affecting subjective health. We operationalised this definition by chronic forms of the diseases anaemia, asthma/chronic obstructive pulmonary disease, atherosclerosis/peripheral arterial occlusive disease, cancer, chronic ischaemic heart disease, chronic low back pain, depression, diabetes mellitus, vertigo, heart failure, osteoarthritis, neuropathy, obesity, osteoporosis, rheumatoid arthritis/chronic polyarthritis and urinary incontinence.

Patients were excluded if (1) they did not meet the criterion for multimorbidity described above; (2) they had been a patient of the practice for less than 12 months or were being treated on behalf of other GPs, e.g. if their practice was currently closed; (3) participation was not recommended for patient safety reasons (according to the GP), e.g. in case of poor health; (4) they lacked capacity to consent; (5) their life expectancy was less than 3 months (according to their GP); (6) they lived in a nursing home; (7) their German language skills were insufficient to participate in the study (according to their GP); (8) they had a severe uncompensated hearing loss and (9) they were participating in other medical studies at the time of recruitment.

Eligible patients received a letter and information material from their GP inviting them to participate in our study. If they were interested, they sent a response letter to the respective study centre. Project staff then contacted the interested patients, explained the study procedure and scheduled an appointment to obtain informed consent and conduct the interview. Recruitment and data collection took place between April 2019 and June 2020.

### Data set

In standardised in-person and telephone interviews, GPs provided information on their age, sex, professional qualifications and experience, and the size and type of their practice. For participating patients from their practice, GPs provided information on diagnoses and course of treatment in order to calculate 24 quality indicators. In the GP interviews, we also documented whether the patients had participated in disease management programmes (DMPs). DMPs are structured programmes for the long-term outpatient management of chronic diseases such as diabetes, coronary heart disease and breast cancer. They involve managed treatment coordinated by the GP, with regular consultations and a focus on patient education and self-management [25, 26].

Patients were visited at home or in their GP practice and were interviewed face-to-face using standardised questionnaires. The questionnaires collected data on sociodemographic characteristics, self-rated health status and health-related quality of life [27], healthcare utilisation, patient satisfaction and the degree of the patients’ commitment to their GP as coordinator of care [28]. Additionally, to calculate 14 quality indicators, patients reported data on the treatment and its outcomes. The data collection and calculation of the indicators are described in Additional file 1.

The patients’ sociodemographic data included age, sex, education level, their living situation and migration background. The education level was based on their general and vocational education and categorised into three levels according to the Comparative Analysis of Social Mobility in Industrial Nations (CASMIN) classification [29], i.e. (1) uncompleted, general elementary or basic vocational education; (2) secondary school certificate or A-level equivalent and (3) tertiary education. Migration background was assessed by the country of birth of the patients and their parents and coded in three categories, i.e. (1) patient and both parents born in Germany; (2) patient born in Germany and at least one parent born abroad and (3) patient born abroad.

The patients’ self-rated health status was rated using the EuroQoL visual analogue scale (EQ-VAS) with values between 0 (worst health status) and 100 (best possible health status). We also measured health-related quality of life using the five-level version of the EuroQol Five-Dimension Scale (EQ-5D-5L). This questionnaire includes the domains mobility, self-care, usual activities, pain or discomfort and anxiety or depression [27]. We computed the EQ-5D-5L index score based on the German value set. This gives a value of 1.000 for full health, which is reduced by up to five subtractions between − 0.026 and − 0.612 depending on the severity of limitations in each of the five domains [30]. In addition, we calculated a morbidity score comprising the number of permanent diagnoses documented in the GP practice.

Utilisation of primary care was assessed through the number of the patients’ contacts with their GP in the previous 3 months. Patient satisfaction was operationalised by asking if patients would recommend their GP to other patients with chronic conditions, which was rated on a four-point Likert scale (‘definitely yes’, ‘rather yes’, ‘rather no’ and ‘definitely no’) and dichotomised for the analyses (‘yes’ vs. ‘no’).

The patients’ willingness to use the GP as coordinator of their treatment was collected using the Questionnaire on Intensity of the Commitment to the GP (‘Fragebogen zur Intensität der Hausarztbindung (F-HaBi)’). The F-HaBi consists of six items rated on a five-point Likert scale and produces a summary score ranging from 0 to 24 points. Higher scores indicate that the patients are more likely to recognise and use the GP as coordinator of their care. Lower scores indicate that the patients prefer to navigate the healthcare system independently [28].

### Statistical analyses

#### Selecting the core sets

Descriptive data were reported as percentages or medians and interquartile ranges (IQR). For both, GP- and patient-reported indicators, a separate summary score was calculated by counting fulfilled indicators at the patient level. In order to obtain a conservative estimate of the quality of care for each patient, we assumed non-fulfilment in case of missing values.

In order to calculate valid summary scores, it was necessary to obtain a one dimensional property of the underlying indicator sets. For each, the GP perspective and the patient perspective, a separate core set of quality indicators was selected by stepwise backward selection based on the corrected item-total correlation of each item [31, 32]. The item-total correlations were calculated by Pearson correlations between the fulfilment status of quality indicators and the Part-Whole-corrected summary score. In each step, the indicator with the lowest item-total correlation among the remaining indicators was excluded. The selection process was continued until all indicators in the remaining indicator set had an item-total correlation of at least r = 0.300 [33–35]. We used our measurement framework [23] as a point of reference to assess whether the key aspects of quality were maintained at the different target levels despite the reduction of items.

#### Assessing the properties of the selected indicators

The selected quality indicators were examined for structural validity, internal consistency, discriminative capacity, feasibility and patient-professional agreement. Structural validity—as indicated by the one-dimensional property of the core sets of quality indicators—was assessed by item-total correlations and exploratory factor analysis. Factors were defined by the principal factors method based on a Pearson correlation matrix and extracted if they had an eigenvalue ≥ 1. Sampling adequacy was determined by the Kaiser–Meyer–Olkin measure.

The discriminative capacity examines whether quality indicators are capable of reflecting meaningful changes in quality of care. Aspects of this measure were the overall fulfilment rates of the indicators as well as the range of performance between providers and floor and ceiling effects, which occur when all patients receiving care from a specific provider are not fulfilling and fulfilling the examined indicator, respectively. Feasibility is given when indicator data can be collected from the specified data sources for a major part of the defined subpopulation. This is reflected in the number of missing values. Moreover, the documentation rate shows if it is possible to obtain data from medical records.

Patient-professional agreement was assessed by agreement between GP and patient perspectives on the performance of the quality indicators. We used positive agreement (PA) and negative agreement (NA) as measures of agreement, which have been shown to be less biased than the more commonly used kappa coefficient [36]. These measures are defined by the formulas

$$PA=\frac{2a}{2a+b+c}\;\mathrm{and}\;NA=\frac{2d}{b+c+2d}$$

with ‘a’ indicating fulfilment in both indicator sources, ‘b’ and ‘c’ indicating fulfilment in one and non-fulfilment in the other indicator source and ‘d’ indicating non-fulfilment in both indicator sources. Cronbach’s alpha was used to assess internal consistency of the selected indicator sets.

#### Analysing construct validity of the quality scores

Multilevel multivariable linear regression models adjusted for random effects at the GP practice level were used to analyse the association between patient characteristics and both summary scores of the selected quality indicators (dependent variables). Independent variables included sociodemographic data, health status, utilisation of primary care, patient satisfaction and the willingness to use the GP as coordinator of treatment. Results from inferential statistics were reported as *ß*-coefficients with 95% confidence intervals (95% CI). An alpha level of 5% (*p* < 0.05) was defined as statistically significant. All statistical analyses were performed using Stata 15.1.

## Results

### Study population

**Fig. 1:**
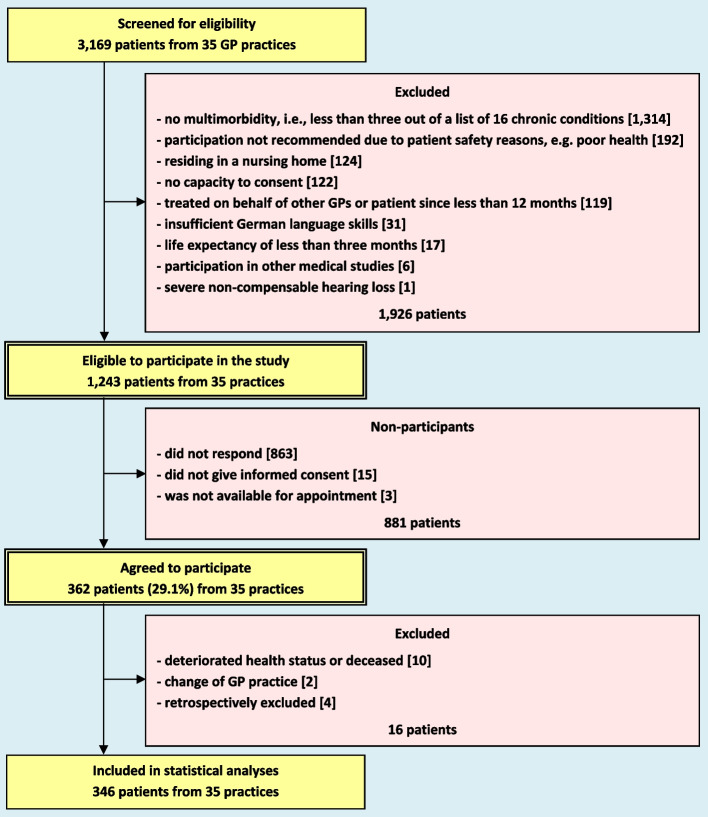
Flow chart of patient recruitment. GP, general practitioner

The recruitment process of study participants is described in Fig. 1. In the participating practices, 1243 eligible patients were contacted for informed consent, 362 patients (29.1%) agreed to participate and 346 could be included in the analyses. The median cluster size was 8 patients per practice (interquartile range: 6 to 13 patients).

Participating GPs had a median age of 57 (IQR 50 to 60) years, and 54.3% were women. GPs had been practising for a median of 20 (IQR 12 to 26) years. More than half of the GP population (57.1%) worked in individual practices, 5.7% in group practices where all physicians have their own patient base, 31.4% in joint practices where all physicians share the same patient base and 5.7% were employed or self-employed in medical care centres. The median number of physicians in the participating practices was 2 (IQR 1–3). In 17.7% of the practices, fewer than 750 patients per quarter were treated, 23.5% of the practices treated 750 to 1000 patients and 58.8% treated 1000 patients per quarter or more.

The patient population is described in Table 1. The patients had a median age of 78 (IQR 72–83) years, and 55.2% were women. More than one third of the patients (35.8%) were living alone. Most patients (56.1%) had uncompleted, general elementary or basic vocational education, and almost nine out of ten patients (88.0%) were born in Germany and had parents, which were also born in Germany. The median number of chronic conditions was 10 (IQR 7–15). The median EQ-5D-5L score was 0.84 (IQR 0.62–0.94) points, and patients rated their health with a median of 65 (IQR 50–80) points. Patients had an average of 2 (IQR 1–3) contacts with their GP in the previous 3 months, 44.6% participated in disease management programmes and nearly nine in ten (89.6%) would recommend their GP to other patients. The median willingness to use their GP as coordinator of treatment was 22 (IQR 19–24) points in the F-HaBi score. As shown in Table 2, the most prevalent diseases in our sample were hypertension (68.2%), chronic low back pain (59.2%) and osteoarthritis (47.3%).

**Table 1:** Patient population

Characteristic	Total (*n* = 346)
Age (in years): median [interquartile range]	78 [72–83]
Sex:	
- Female	55.2%
- Male	44.8%
Living situation: living alone	35.8%
Education (pursuant to CASMIN):	
- Uncompleted, general elementary or basic vocational	56.1%
- Secondary school certificate or A-level equivalent	27.9%
- Tertiary education	16.0% (*n* = 344)
Migration background:	
- Patient and both parents born in Germany	88.0%
- Patient born in Germany and at least one parent abroad	4.4%
- Patient born abroad	7.6% (*n* = 342)
Number of chronic conditions (39 categories): median [interquartile range]	10 [7–15]
Health-related quality of life (pursuant to EQ-5D-5L, German value set): median [interquartile range]	0.84 [0.62–0.94] (*n* = 337)
Self-rated Health (visual analogue scale): median [interquartile range]	65 [50–80] (*n* = 340)
Number of contacts with GP (last 3 months): median [interquartile range]	2 [1–3] (*n* = 339)
Enrolment in a disease management programme	44.6% (*n* = 343)
Patient satisfaction (willingness to recommend the GP):	
- “Definitely no” or “rather no”	10.4%
- “Definitely yes” or “rather yes”	89.6% (*n* = 336)
Willingness to use GP as coordinator of treatment (pursuant to F-HaBi): median [interquartile range]	22 [19–24] (*n* = 340)

^a^Low quality score: median (= 8 points) or less

^b^Low quality score = higher than median (= 8 points)

*n*, number of participants; *p*, probability value; *CASMIN*, Comparative Analysis of Social Mobility in Industrial Nations; *EQ-5D-5L*, EuroQoL five dimension five level scale; *GP*, general practitioner; *F-HaBi*, *Fragebogen zur Intensität der Hausarztbindung* (‘Questionnaire on Intensity of the Commitment to the GP’)

**Table 2:** Chronic conditions with sample prevalence ≥ 5%

Chronic conditions	Prevalence in the sample (*n* = 346)
Hypertension	68.2%
Chronic low back pain^a^	59.2%
Osteoarthritis^a^	47.4%
Diabetes mellitus^a^	40.8%
Dyslipidaemia	35.3%
Chronic ischaemic heart disease^a^	34.1%
Asthma/chronic obstructive pulmonary disease^a^	29.2%
Cancer^a^	29.2%
Depression^a^	27.8%
Thyroid dysfunction	24.9%
Chronic gastritis/gastro-oesophageal reflux disease	24.6%
Atherosclerosis/peripheral arterial occlusive disease^a^	21.7%
Cardiac arrhythmia	20.5%
Osteoporosis^a^	19.1%
Neuropathy^a^	18.8%
Obesity^a^	17.6%
Heart failure^a^	17.3%
Urinary incontinence^a^	15.3%
Varicose veins of lower extremities	13.9%
Renal failure	13.3%
Intestinal diverticulosis	11.6%
Heart valve disease	10.1%
Severe visual impairment	10.1%
Cerebral ischaemia/chronic stroke	9.5%
Rheumatoid arthritis/chronic polyarthritis^a^	8.7%
Vertigo^a^	8.1%
Somatoform disorders	7.8%
Insomnia	7.8%
Hyperuricemia/gout	7.5%
Liver disease	6.4%
Prostatic hyperplasia	5.5%
Chronic cholecystitis/gallstones	5.2%

^a^Inclusion criterion

### Selection of the core sets

The stepwise backward selection to define the core sets of quality indicators is detailed in the Tables 3 and 4. Twelve GP-assessed quality indicators and seven patient-assessed quality indicators were excluded. In the previous stages of the project, a measurement framework for healthcare quality [23] had been proposed. Table 5 shows that all three levels of healthcare and all nine care domains of the complete indicator set are represented by both core sets combined. The GP-reported indicators cover eight domains and the patient-reported indicators cover five domains. The final questionnaires for both core sets can be found in Additional file 2.

**Table 3:** Excluded quality indicators (GP assessment)

Step	Item-total correlation	Quality indicator	Performance	Missing values
1	< 0.001	Assessment of biopsychosocial support needs according to ICF^a^ (*n* = 325)	0%	6.1%
2	0.036	Identification of patients with multimorbidity (*n* = 345)	18.0%	0.3%
3	0.097	Documentation of adverse drug reactions (*n* = 35)	38.2%	2.9%
4	0.157	Addressing financial support needs (*n* = 325)	19.1%	7.5%
5	0.168	Information about medication (*n* = 333)^b^	98.8%	1.5%
6	0.188	Regular updates of medication plan (*n* = 301)^c^	52.2%	2.6%
7	0.179	Assessment of symptom burden (*n* = 333)	14.7%	3.8%
8	0.204	Screening for depression (*n* = 266)^d^	13.2%	8.0%
9	0.254	Written treatment plan (*n* = 330)	9.1%	4.6%
10	0.248	Facilitating patient education and self-management (*n* = 285)	23.9%	17.6%
11	0.250	Comprehensive care documentation (*n* = 344)	81.4%	0.6%
12	0.267	Assigning responsibility for coordination of care (*n* = 333)	28.8%	3.8%

^a^ICF = International Classification of Functioning, Disability and Health [37]

^b^Excluding patients without any medication (*n* = 8)

^c^Excluding patients with less than 3 different regular medications (*n* = 36)

^d^Excluding patients with prior diagnosis of depression (*n* = 57)

**Table 4:** Excluded quality indicators (patient assessment)

Step	Item-total correlation	Quality indicator	Performance	Missing values
1	0.136	Written treatment plan (*n* = 338)	6.5%	2.3%
2	0.123	Facilitating patient education and self-management (*n* = 325)	40.9%	6.1%
3	0.163	Regular updates of medication plan (*n* = 315)^a^	74.0%	0.6%
4	0.186	Addressing financial support needs (*n* = 333)	6.3%	3.8%
5	0.197	Shared decision-making (*n* = 330)	74.5%	4.6%
6	0.236	Involving partner, family and caregivers (*n* = 336)	43.5%	2.9%
7	0.271	Information about potential benefits and harms of treatment options (*n* = 183)	63.4%	47.1%

^a^Excluding patients with less than 3 different regular medications (*n* = 29)

**Table 5:** Levels of healthcare and care domains of selected core sets and excluded quality indicators

Target level of health care	Care domains	Quality indicator	Representation
Patient factors	Physical and mental health	Proactive pain assessment	**GP and patient report**
		*Screening for depression*	*Excluded*
		*Identification of patients with multimorbidity*	*Excluded*
	Personal background and living environment	Involving partner, family and caregivers	**GP report**
		*Addressing financial support needs*	*Excluded*
	Coping and skills	Monitoring adherence to treatment	**GP report**
		*Facilitating patient education and self-management*	*Excluded*
	Quality of life	Quality of life assessment	**GP report**
		*Assessment of symptom burden*	*Excluded*
		*Assessment of biopsychosocial support needs according to ICF*	*Excluded*
	Patient preferences	Eliciting patient preferences	**GP and patient report**
Patient-provider communication	Information and decision-making	Mutual agreement on treatment goals	**GP and patient report**
		Information about potential benefits and harms of treatment options	**GP report**
		Information about medication	**Patient report**
		*Shared decision making*	*Excluded*
	Care planning and clinical management	Assessment of treatment burden	**GP and patient report**
		Medication review	**GP and patient report**
		Monitoring of pain management	**GP report**
		*Written treatment plan*	*Excluded*
		*Documentation of adverse drug reactions*	*Excluded*
		*Regular updates of medication plan*	*Excluded*
Context and organisational structures	Coordination	Assigning responsibility for coordination of care	**Patient report**
		*Comprehensive care documentation*	*Excluded*
	Training	Training of physician staff addressing multimorbidity	**GP report**
		Training of non-physician staff addressing multimorbidity	**GP report**

Excluded quality indicators are in italic letters

*ICF* International Classification of Functioning, Disability and Health

### Properties of the selected indicators

The characteristics of the core quality indicators are presented in Table 6. The GP-reported indicators had an item-total correlation between 0.332 and 0.576 and the patient-reported indicators between 0.339 and 0.440. Both exploratory factor analyses resulted in one extracted factor with an eigenvalue of 3.27 and 1.33, respectively. The core quality indicators had loadings between 0.416 and 0.673, and 0.311 and 0.545, respectively. The Kaiser–Meyer–Olkin measure of sampling adequacy was 0.774 and 0.758, respectively. We determined a Cronbach’s alpha of 0.806 and 0.628, respectively, for the selected core indicator sets.

**Table 6:** Characteristics of selected core sets of quality indicators

	**Validity**		**Discriminative capacity**			**Feasibility**		**Patient-professional agreement**	
	**Item-total**	**Factor loadings**^**a**^	**Performance**	**Floor effects**	**Ceiling effects**	**Documentation**	**Missing values**	**Positive**	**Negative**
**GP-reported quality indicators**									
Eliciting patient preferences (*n* = 323)	0.576	0.673	46.1%	11.8%	5.9%	69.1%	6.6%	49.4%	61.0%
Quality of life assessment (*n* = 321)	0.512	0.643	80.1%	2.9%	28.6%	60.3%	7.2%	-^g^	-^g^
Mutual agreement on treatment goals (*n* = 313)	0.501	0.612	48.6%	11.4%	2.9%	73.0%	9.5%	54.1%	54.4%
Assessment of treatment burden (*n* = 308)	0.435	0.613	75.0%	2.9%	31.4%	57.0%	11.0%	43.5%	42.8%
Medication review (*n* = 295)^b^	0.423	0.476	39.0%	41.2%	11.8%	62.3%	12.7%	54.1%	43.4%
Training of non-physician staff addressing multimorbidity (*n* = 35)^c^	0.416	0.479	60.6%	-^e^	-^e^	-^f^	5.7%	-^g^	-^g^
Monitoring adherence to treatment (*n* = 313)	0.387	0.474	47.0%	14.7%	8.8%	50.3%	9.5%	-^g^	-^g^
Monitoring of pain management (*n* = 203)^d^	0.385	0.430	67.0%	5.9%	26.5%	65.4%	1.5%	-^g^	-^g^
Involving partner, family and caregivers (*n* = 317)	0.376	0.431	33.8%	35.3%	5.9%	72.0%	8.4%	36.1%	59.8%
Training of physician staff addressing multimorbidity (*n* = 35)^b^	0.372	0.438	71.4%	-^e^	-^e^	-^f^	0%	-^g^	-^g^
Proactive pain assessment (*n* = 324)	0.335	0.423	62.3%	2.9%	17.1%	69.8%	6.4%	47.8%	53.2%
Information about benefits and harms of treatment options (*n* = 318)	0.332	0.488	89.0%	2.9%	34.3%	59.2%	8.1%	75.9%	24.1%
**Patient-reported quality indicators**									
Eliciting patient preferences (*n* = 323)	0.440	0.535	41.2%	11.4%	2.9%	-^i^	6.6%	49.4%	61.0%
Assessment of treatment burden (*n* = 329)	0.370	0.459	24.6%	28.6%	0%	-^i^	4.9%	43.5%	42.8%
Information about medication (*n* = 227)^h^	0.362	0.311	84.1%	0%	91.4%	-^i^	12.4%	90.7%	5.1%
Mutual agreement on treatment goals (*n* = 334)	0.348	0.446	50.3%	8.6%	5.7%	-^i^	3.5%	54.1%	54.4%
Proactive pain assessment (*n* = 327)	0.345	0.436	30.9%	14.3%	0%	-^i^	5.5%	47.8%	53.2%
Medication review (*n* = 336)^b^	0.343	0.416	69.1%	2.9%	5.7%	-^i^	0.9%	54.1%	43.4%
Assigning responsibility for coordination of care (*n* = 332)	0.339	0.421	44.0%	11.4%	0%	-^i^	4.0%	33.3%	61.8%

^a^Results from explorative factor analysis with one extracted factor (eigenvalue 3.48)

^b^Excluding patients without medication (*n* = 8)

^c^Measured on GP level

^d^Excluding patients without chronic pain (*n* = 138)

^e^Ceiling effects are equal to the rate of fulfilment and floor effects are equal to rate of non-fulfilment

^f^Due to indicator definition, documentation rate is 100%

^g^No patient indicator defined

^h^Excluding patients without continuous medication (*n* = 6) and if medication was prescribed by specialist (*n* = 81)

^i^Not assessed for patient-reported indicators

*GP*, general practitioner; *n*, number of participants

Overall, the fulfilments rate of the indicators ranged from 24.6 to 89.0%. Fourteen quality indicators had floor effects between 0 and 14.7%, and the others were at 28.6%, 35.3% and 41.2%. Eleven indicators had ceiling effects between 0 and 11.8%, five were between 17.1 and 34.3% and one at 91.4%. For the ten analysed indicators, documentation rates ranged from 50.3 to 73.0%. Between 0 and 12.7% of the values were missing. With positive agreement rates between 33.3 and 75.9% and negative agreement rates between 24.1 and 61.8%, seventeen of the analysed indicators showed low to moderate agreement between patients and professionals. One indicator had 90.7% positive agreement and 5.1% negative agreement.

### Construct validity of the quality scores

The results of the multivariable analyses of the associations between patient characteristics and GP- and patient-reported quality scores are shown in the Tables 7 and 8. The GP-reported quality score was lower when patients were born abroad (− 1.04, 95% CI − 2.00/ − 0.08, *p* = 0.033) and when they had higher health-related quality of life (− 1.37 per point in the EQ-5D-5L score, 95% CI − 2.39/ − 0.36, *p* = 0.008). The quality score was higher when the patients had more contacts with their GP (0.14 per contact, 95% CI 0.04/0.23, *p* = 0.007) and when they were more willing to use the GP as coordinator of treatment (0.13 per point F-HaBi score, 95% CI 0.06/0.20, *p* < 0.001). The patient-reported quality score was higher when patients visited their GP more often (0.12 per contact, 95% CI 0.04/0.20, *p* = 0.006) and when they had a higher level of commitment to their GP (0.16 per point, 95% CI 0.10/0.21, *p* < 0.001).

**Table 7:** Association between patient characteristics and GP-reported quality score (*n* = 306)

Characteristic	*ß* (95% CI)	*p*
Age (per 10 years)	0.12 (− 0.23/0.48)	0.500
Sex:		
- Women	Reference	
- Men	0.08 (− 0.44/0.60)	0.763
Living situation		
- Living together with others	Reference	
- Living alone	0.29 (− 0.23/0.81)	0.275
Educational level (pursuant to CASMIN):		
- Uncompleted, general elementary or basic vocational	Reference	
- Secondary school certificate or A-level equivalent	− 0.08 (− 0.63/0.48)	0.785
- Tertiary education	0.09 (− 0.64/0.82)	0.808
***Migration background:***		
***- Patient and both parents born in Germany***	***Reference***	
- Patient born in Germany and at least one parent abroad	− 0.58 (− 1.77/0.61)	0.339
***- Patient born abroad***	− ***1.04 (− 2.00/ − 0.08)***	***0.033***
Number of chronic conditions	0.05 (− 0.02/0.11)	0.167
Self-rated health (visual analogue scale, per 10 points)	0.05 (− 0.10/0.19)	0.505
***Health-related quality of life (pursuant to EQ-5D-5L, German value set)***	*** − 1.37 (− 2.39/ − 0.36)***	***0.008***
***Number of contacts with GP (last 3 months)***	***0.14 (0.04/0.23)***	***0.007***
Enrolment in a disease management programme	0.52 (0.00/1.05)	0.050
Patient satisfaction: willingness to recommend the GP	− 0.71 (− 1.54/0.12)	0.094
***Willingness to use GP as coordinator of treatment (pursuant to F-HaBi)***	***0.13 (0.06/0.20)***	** < *0.001***

*ß*, coefficient; *95% CI*, 95% confidence interval; *p*, probability value; *CASMIN*, Comparative Analysis of Social Mobility in Industrial Nations; *EQ-5D-5L*, EuroQoL five dimension five level scale; *GP*, general practitioner; *F-HaBi*, *Fragebogen zur Intensität der Hausarztbindung* (“Questionnaire on Intensity of the Commitment to the GP”)

**Table 8:** Association between patient characteristics and patient-reported quality score (*n* = 306)

Characteristic	*ß* (95% CI)	*p*
Age (per 10 years)	− 0.12 (− 0.40/0.16)	0.391
Sex:		
- Women	Reference	
- Men	0.03 (− 0.38/0.44)	0.880
Living situation		
- Living together with others	Reference	
- Living alone	0.33 (− 0.08/0.73)	0.116
Educational level (pursuant to CASMIN):		
- Uncompleted, general elementary or basic vocational	Reference	
- Secondary school certificate or A-level equivalent	0.11 (− 0.32/0.54)	0.623
- Tertiary education	0.01 (− 0.55/0.58)	0.960
Migration background:		
- Patient and both parents born in Germany	Reference	
- Patient born in Germany and at least one parent abroad	− 0.24 (− 1.18/0.71)	0.623
- Patient born abroad	0.41 (− 0.35/1.16)	0.290
Number of chronic conditions	0.03 (− 0.01/0.06)	0.125
Self-rated Health (visual analogue scale, per 10 points)	− 0.06 (− 0.18/0.05)	0.289
Health-related quality of life (pursuant to EQ-5D-5L, German value set)	0.16 (− 0.63/0.96)	0.686
***Number of contacts with GP (last 3 months)***	***0.12 (0.04/0.20)***	***0.002***
Enrolment in a disease management programme	− 0.25 (− 0.64/0.14)	0.211
Patient satisfaction: willingness to recommend the GP	0.46 (− 0.19/1.11)	0.162
***Willingness to use GP as coordinator of treatment (pursuant to F-HaBi)***	***0.16 (0.10/0.21)***	** < *0.001***

*ß*, coefficient; *95% CI*, 95% confidence interval; *p*, probability value; *CASMIN*, Comparative Analysis of Social Mobility in Industrial Nations; *EQ-5D-5L*, EuroQoL five dimension five level scale; *GP*, general practitioner; *F-HaBi*, *Fragebogen zur Intensität der Hausarztbindung* (“Questionnaire on Intensity of the Commitment to the GP”)

## Discussion

### Statement of principal findings

To our knowledge, the MULTIqual project is the first study to develop and validate quality indicators for the primary care of patients with multimorbidity in a systematic, multi-step approach. In this study, we selected core sets of twelve GP-reported and seven patient-reported quality indicators that represented all nine care quality dimensions of the complete indicator set, demonstrated good internal consistency and robust structural and construct validity and can be collected through new or already existing GP and patient surveys. Depending on access to data sources, either patient-reported or GP-reported—or both indicator core sets—can be used, allowing for broader application of the indicators. The core sets provide viable alternatives to the untested set of indicators, as the size of the set and the cost of measurement are also important considerations for implementation [38–40].

### Strengths and limitations

Following a well-established methodology, our quality indicators were identified through a multistep process that combined available evidence and expert consensus [41], represent multiple systematically selected domains of care specific for populations with multimorbidity and are therefore a more valid alternative to the fragmented and disease-specific quality assessment through existing patient-reported experience and outcome measures [15] such as or the European Task Force on Patient Evaluation of General Practice Care questionnaire (EUROPEP) [42] or EQ-5D [27]. As the indicators were developed based on literature review and expert opinions without connection to a specific disease spectrum [23], our indicators are per se generic and equally applicable to all multimorbidity constellations that impact subjective health status.

Data were analysed using multivariable analyses, adjusted for potential confounders, and multilevel models, allowing for cluster effects. However, the core sets of quality indicators were selected by a backward selection algorithm, which is known to be sensitive to differences in the distribution of the included variables. As a result, the identified core sets represent coherent sets of quality indicators, but not necessarily the best possible selection. It is important to note that the data in our study were collected via self-report, which may introduce recall problems, errors and social desirability. The study design was cross-sectional, which means that the direction of associations cannot be determined, i.e. whether quality scores influence quality of life or vice versa.

The response rate for this study was a low (29.1%), possibly limiting its representativeness as certain groups (e.g. male, younger, less educated and less healthy living patients) are often underrepresented in low-response samples. Despite these differences in descriptive data between samples and the population, there is usually little effect of low response rates on the reported associations in the dataset [43–46]. For patient safety reasons, we had to exclude patients in poor health, which narrows our construct of multimorbidity. Furthermore, only diseases that are frequently co-occurring with other diseases [47, 48] were defined as inclusion criteria. However, many common diseases that do not fall within this definition are still prevalent in our sample such as chronic gastritis/gastroesophageal reflux disease (24.6%) or liver disease (6.4%), as patients with additional conditions were not excluded.

Another potential bias in our study sample is that participating GPs are likely to be highly motivated and interested in the topic. The quality of primary care for patients with multimorbidity might therefore be overestimated. Moreover, the study was conducted in major German cities with a high density of healthcare providers. The average population of German GPs is slightly younger than our study sample (55 vs. 57 years), the proportion of women is slightly lower (49% vs. 54%) and on average practices are smaller (average of 850 patients per quarter vs. 59% treating 1000 patients or more [49]). Therefore, caution should be taken when generalising the results to medically deprived areas.

Finally, it should be mentioned that this study was observational and had multiple outcomes without prior sample size calculation. Due to the relatively small sample size of 346 patients from 35 practices, predictors of reduced healthcare quality may have been missed due to limited statistical power.

### Comparison with the literature

Pilot testing of quality indicators for primary care and community settings is rarely reported. Consequently, well-defined criteria and standards for empirical validation are lacking [50, 51]. In Germany, testing of quality indicators is mainly carried out by central organisations commissioned by the Federal Joint Committee [21]. This makes the application and validation of core sets by independent researchers and health experts an innovative component of the MULTIqual project.

Previous studies have shown that quality of care increases with the number of diagnoses when using disease-specific indicators, particularly in concordant conditions with similar pathophysiological profiles and disease management [52–54]. However, patient safety and patient-centred outcomes have been found to be negatively associated with the number and severity of conditions [54, 55]. There is evidence linking higher severity of comorbidities with higher quality of care according to process measures. This is consistent with our findings of an inverse relationship between quality of life and quality of care per GP-reported measures [54]. Zulman et al. [56] hypothesise that higher healthcare utilisation by patients, e.g. due to clinical complexity and reduced health status, leads to more intensive monitoring, more frequent assessment of healthcare needs and subsequent adjustments to their treatment.

In our study, quality scores improve with commitment to the GP. In Germany, which does have a compulsory primary care system and allows free choice of healthcare provider, this relationship is based solely on mutual trust and voluntariness [57]. However, we did not find evidence supporting the link between participation in DMPs and improvements in care structure and processes [25, 58, 59], although with enrolment in a DMP, some of the criteria measured by the indicator sets should already become an integral part of the care regimen.

### Implications for research and clinical practice

The results of the pilot study demonstrated that the core sets can be a useful tool for the identification of areas in primary care with potential for improvement. Although many researchers advocate for patient-centred care in the context of multimorbidity [60–62], treatment goals or patient preferences were established in less than half of all cases. These findings suggest that patient-centred care planning is not yet fully realised. Tinetti et al. [63] were able to show that aligning care with patient preferences led to a reduction in unwanted treatments, medications and diagnostic tests. Widespread adoption of these principles could potentially have a similar impact on the German healthcare system, where patients with multimorbidity incur significant healthcare utilisation due to the lack of gatekeeping in primary care [4, 64].

Indicators of process quality are most useful for quality improvement purposes as they more directly reflect changes in practice [65, 66]. Moreover, our findings may guide the future development of electronic documentation systems, ultimately seeking to improve documentation quality and enable quality monitoring through built-in performance measurement [67]. In Germany, the digital transformation of GP practices is still in its early stages, so that despite major barriers to this development, further progress in current documentation standards can be expected in the coming years [68, 69].

While the development of the candidate indicator was informed by international evidence—most notably the multimorbidity guideline by the UK National Institute for Health and Care Excellence [70], the German College of General Practitioners [71] and the American Geriatrics Society [72]—evaluation and consensus of the indicators was obtained by a German expert panel and is thus geared to the specifics of the German healthcare system [23]. Therefore, in principle, the indicators are internationally relevant and transferable to other healthcare systems. Nevertheless, it will be necessary to adapt indicator descriptions and modes of data collection. In particular, it should be examined if easily accessible data can be used as data sources for quality indicators [73, 74], e.g. standardised documentation in medical records in the UK [75].

Longitudinal studies are required to examine the responsiveness of quality scores to change, costs and potential unintended consequences, as well as long-term benefits resulting from the implementation of these quality indicators. This should be done by conducting a cost-utility analysis and measuring changes in indicator scores over time in relation to health outcomes [21, 76, 77]. Unfortunately, there is still no robust evidence of the benefits of using quality indicators. However, improvements in care processes have been achieved by creating the conditions for the implementation of indicators, including increased use of digital solutions, prompts, recall systems and better documentation [78]. In light of future advances in multimorbidity research and corresponding changes in guideline recommendations, the indicators should be regularly updated to best reflect current evidence [79].

## Conclusions

The quality indicator core sets developed in our study are the first brief measurement tools specifically designed to assess the quality of care for people with multimorbidity. Our results demonstrate that development and validation of such indicators for multimorbidity are feasible and can be extended to other countries. By offering a viable alternative to disease-specific metrics, the core sets can facilitate the implementation of treatment standards, promote patient-centred care and provide guidance for the future development of electronic documentation systems. However, further research is necessary to understand the cost–benefit ratio of implementing these indicators.

## Supplementary Information

**Additional file 1.** Data collection and calculation of the indicators.

**Additional file 2.** Questionnaires.

## Data Availability

The datasets generated and analysed during the current study are not publicly available, because the patient consent statement did not specify that data would be published, but are available from the corresponding author on reasonable request.

## Abbreviations

95% CI: 95% Confidence intervals
CASMIN: Comparative Analysis of Social Mobility in Industrial Nations
DMPs: Disease management programmes
EUROPEP: European Task Force on Patient Evaluation of General Practice Care
EQ-5D-5L: EuroQol Five-Dimension Scale
EQ-VAS: EuroQoL visual analogue scale
F-HaBi: Questionnaire on Intensity of the Commitment to the GP (‘Fragebogen zur Intensität der Hausarztbindung’)
GP: General practitioner
ICF: International Classification of Functioning, Disability and Health
IQR: Interquartile range
*n*: Number of participants
NA: Negative agreement
*p*: Probability value
PA: Positive agreement
