# Supplementary material for: Validation of patient- and GP-reported core sets of quality indicators for older adults with multimorbidity in primary care: results of the cross-sectional observational MULTIqual validation study
Source: BMC Med. 2023 Apr 17;21:148. doi: 10.1186/s12916-023-02856-0 (PMC10111827; doi:10.1186/s12916-023-02856-0)
Supplement: Supplementary file 1 — Additional file 1. Data collection and calculation of the indicators. [file 12916_2023_2856_MOESM1_ESM.pdf]

VALIDATION OF PATIENT- AND GP-REPORTED CORE SETS OF QUALITY INDICATORS FOR OLDER ADULTS WITH MULTIMORBIDITY IN PRIMARY CARE: RESULTS OF THE CROSS-SECTIONAL OBSERVATIONAL MULTIQUAL VALIDATION STUDY

Additional file 1: Data collection and calculation of the indicators

Indicator: Proactive pain assessment

Specification

Description: Proportion (%) of patients aged 65 and over with three or more chronic conditions who were asked about the presence of pain

Target value: Maximum possible

Rationale: Many people with multimorbidity suffer from chronic pain. This can have a strong impact on mental well-being and the ability to function in everyday life. Chronic pain also increases the risk of falls and the development of anxiety (German College of General Practitioners and Family Physicians 2017). Although it is often assumed that pain is actively addressed by the patient in the consultation, the prevalence rates for pain problems are much higher than the rate of pain as a reason for consultation in GP practices suggests. A significant proportion of pain problems are therefore not recorded (German College of General Practitioners and Family Physicians 2021).

Indicator type: Process quality

Quality dimension: Appropriateness of care, timeliness and accessibility of care, patient safety

Calculation of the indicator

Reference period: Three months

Data collection: GP survey/documentation of presence of in medical records

Numerator: Number of patients who were asked about the presence of pain

Denominator: Number of patients aged 65 and over with three or more chronic conditions

Further inclusion and exclusion criteria: None

Indicator question(s): Has your GP asked you specifically about the presence of pain in the last three months?

□ Yes □ No □ Not sure

Previous use and evidence

Underlying recommendation:

NICE Multimorbidity: clinical assessment and management (2016), p. 237: "Be alert to the possibility of (...) chronic pain and the need to assess this and the adequacy of pain management" *(limited support for the recommendation)*

Original indicator: Not available

Indicator: Involving partners, family and caregivers

Specification

Description: Proportion (%) of patients aged 65 and over with three or more chronic conditions that had a discussion whether and to what extent partners, family and caregivers should be involved in important decisions

Target value: Maximum possible

Rationale: Patients often want family members, friends or caregivers to be involved in decision-making. Especially for older patients, the support of these persons plays an important role in their care (National Institute for Health and Care Excellence 2016). It may even be desired that these caregivers make decisions for the patient, especially in the presence of cognitive impairment (American Geriatrics Society Expert Panel on the Care of Older Adults with Multimorbidity 2012).

Indicator type: Process quality

Quality dimension: Patient-centeredness

Calculation of the indicator

Reference period: At least once per patient and incident-related (upon new diagnosis, deterioration of health status, hospital admission/discharge)

Data collection: GP survey/documentation of involvement of partners, friends and caregivers in medical records

Numerator: Number of patients that had a discussion whether and to what extent partners, family and caregivers should be involved in important decisions

Denominator: Number of patients aged 65 and over with three or more chronic conditions

Further inclusion and exclusion criteria: None

Indicator question(s): Has it been discussed with the patient in general whether and to what extent partners, family and caregivers should be involved in important decisions regarding their care?

□ Yes □ No □ Not sure

What was the last significant change in the patient's health status?
(Note: Several may apply)

□Deterioration of health status □ Improvement in health status
□Hospitalisation □New diagnosis
□ Other: ________________________

On this occasion, has it been discussed again whether and to what extent partners, family and caregivers should be involved in important decisions about their care?

□ Yes □ No □ Not sure

Previous use and evidence

Underlying recommendation:

NICE Multimorbidity: clinical assessment and management (2016), p. 240: "Clarify with the patient whether and how they would like their partner, family members and/or carers to be involved in key decisions about the management of their conditions. Review this regularly. If the patient agrees, share information with their partner, family members and/or carers" *(strong support for the recommendation)*

DEGAM Multimorbidity S3 Guideline (2017), p. 23: "It should be clarified with the patient whether and to what extent partners, relatives or carers should be involved in important care decisions" *(moderate support for the recommendation)*

Original indicator: Not available

Indicator: Monitoring adherence to treatment

Specification

Description: Proportion (%) of patients aged 65 and over with three or more chronic conditions whose adherence to treatment was assessed

Target value: Maximum possible

Rationale: Adherence is one of the most important modifiable factors influencing treatment outcome across all conditions (World Health Organization 2003). Due to the large number of possible health-related tasks, adherence is central to the success of treatment in patients with multimorbidity.

Notes: Adherence is the extent to which a patient's behaviour is consistent with treatment goals and pathways previously agreed upon with the physician or therapist (World Health Organization 2003).

Indicator type: Process quality

Quality dimension: Patient safety, effectiveness of care

Calculation of the indicator

Reference period: 12 months

Data collection: GP survey/documentation of adherence in medical records

Numerator: Number of patients whose adherence to treatment was assessed

Denominator: Number of patients aged 65 and over with three or more chronic conditions

Further inclusion and exclusion criteria: None

Indicator question(s): Did you have a discussion with the patient about their adherence to treatment?

□ Yes, within the last 12 months □ Yes, more than 12 months ago
□ No □ Not sure

Previous use and evidence

Underlying recommendation:

American Geriatrics Society Expert Panel on the Care of Older Adults with Multimorbidity (2012), p. 11: "Consider treatment complexity and feasibility when making clinical management decisions for older adults with multimorbidity. (...) Because treatment complexity often increases with multimorbidity, an interdisciplinary team should assess the ability of older adults with multimorbidity to manage or adhere to a treatment plan or medication regimen on an ongoing basis" *(strength of recommendation not available)*

Original indicator: Not available

Indicator: Quality of life assessment

Specification

Description: Proportion (%) of patients aged 65 and over with three or more chronic conditions that had a discussion of their subjective quality of life

Target value: Maximum possible

Rationale: The improvement of quality of life is a central focus of patient-centred treatment in multimorbidity (German College of General Practitioners and Family Physicians 2017).

Indicator type: Process quality

Quality dimension: Patient-centeredness

Calculation of the indicator

Reference period: 12 months

Data collection: GP survey/documentation of discussion about quality of life in medical records

Numerator: Number of patients that had a discussion of their subjective quality of life

Denominator: Number of patients aged 65 and over with three or more chronic conditions

Further inclusion and exclusion criteria: None

Indicator question(s): Did you have a discussion with the patient about their perceived quality of life?

□ Yes, within the last 12 months □ Yes, more than 12 months ago
□ No □ Not sure

Previous use and evidence

Underlying recommendation:

NICE Multimorbidity: clinical assessment and management (2016), p. 237: “Establish disease burden by talking to people about how their health problems affect their day-to-day life. Include a discussion of (...) how health problems interact and how this affects quality of life” *(strong support for the recommendation)*

Original indicator: Not available

Indicator: Eliciting patient preferences

Specification

Description: Proportion (%) of patients aged 65 and over with three or more chronic conditions whose priorities, goals and values were discussed and documented

Target value: Maximum possible

Rationale: Patient-centred care and treatment planning should be based on individual preferences and goals. Personal values and priorities determine how impairments due to chronic conditions are perceived and evaluated and therefore also determine individual support needs (National Institute for Health and Care Excellence 2016).

Indicator type: Process quality

Quality dimension: Patient-centeredness, participation and active involvement of the patient

Calculation of the indicator

Reference period: Three months

Data collection: GP survey/documentation of patient preferences in medical records, patient survey

Numerator: Number of patients whose priorities, goals and values were discussed and documented

Denominator: Number of patients aged 65 and over with three or more chronic conditions

Further inclusion and exclusion criteria: None

Indicator question(s): GP survey:
Did you have a discussion with the patient about their preferences (values, goals and priorities) regarding their health care?
(Note: Preferences may include, e.g., maintaining employment, participating in social activities/family life, preventing adverse events (e.g., stroke), minimising adverse effects of medications, reducing burden of treatments, prolonging life.)

□ Yes, within the last three months □ Yes, within the last 12 months
□ Yes, more than 12 months ago □ No
□ Not sure

Patient survey:

Thinking about the last three months, has your GP asked you about your wishes, health goals and what is important to you in your treatment?

(Note: Examples may include participation in social activities, family life, prevention of negative events such as strokes or falls, minimisation of medication side effects, pain reduction, etc.)

□ Yes □ No □ Not sure

Previous use and evidence

Underlying recommendation:

NICE Multimorbidity: clinical assessment and management (2016), p. 240: "Encourage people with multimorbidity to clarify what is important to them, including their personal goals, values and priorities. These may include maintaining their independence, undertaking paid or voluntary work, taking part in social activities and playing an active part in family life, preventing specific adverse outcomes (for example, stroke), reducing harms from medicines, reducing treatment burden, lengthening life" *(strong support for the recommendation)*

DEGAM Multimorbidity S3 Guideline (2017), p. 23: "When identifying patient preferences and values, the following aspects should be addressed (adapted from NICE guideline): Patients should be encouraged to state their personal goals and priorities. This includes clarifying the importance of: Maintaining their social role in work/occupation, participation in social activities, family life; preventing specific events (e.g., stroke); minimising medication side effects; reducing burden of treatments; prolonging life" *(strong support for the recommendation)*

Original indicator: *NICE Multimorbidity quality standard (2017), p. 9:* "Proportion of adults with an individualised management plan for multimorbidity whose plan has a record of values, priorities and goals"

Indicator: Mutual agreement on treatment goals

Specification

Description:

Proportion (%) of patients aged 65 and over with three or more chronic conditions with whom treatment goals were established in mutual agreement

Target value: Maximum possible

Rationale: Patients with multimorbidity and GPs often set different priorities in treatment, which is why a negotiation process is needed to define care goals (German College of General Practitioners and Family Physicians 2017).

Indicator type: Process quality

Quality dimension: Patient-centeredness, effectiveness of care

Calculation of the indicator

Reference period: Three months

Data collection: GP survey/documentation of treatment goals in medical records, patient survey

Numerator: Number of patients with whom treatment goals were established in mutual agreement

Denominator: Number of patients aged 65 and over with three or more chronic conditions

Further inclusion and exclusion criteria: None

Indicator question(s): GP survey

Did you agree on treatment goals together with the patient?

□ Yes, within the last three months □ Yes, within the last 12 months
□ Yes, more than 12 months ago □ No
□ Not sure

Patient survey

Thinking about the last three months, have you discussed your treatment goals with your GP?

□ Yes □ No □ Not sure

Previous use and evidence

Underlying recommendation:

NICE Multimorbidity: clinical assessment and management (2016), p. 99: "Agree an individualised management plan with the person including (...) goals and plans for future care" *(strong support for the recommendation)*

DEGAM Multimorbidity S3 Guideline (2017), p. 37: "Any decision should be made against the background of the patient's preferences, which often only develop in discussion, and the joint prioritisation of treatment goals" *(strong support for the recommendation)*

Original indicator: Not available

Indicator: Information on potential benefits and harms of treatment options

Specification

Description: Proportion (%) of patients aged 65 and over with three or more chronic conditions who were informed about potential benefits and risks of treatment options prior to treatment decisions

Target value: Maximum possible

Rationale: Patients and physicians may have different perspectives on potential benefits and harms of treatments and may assess their relevance differently (American Geriatrics Society Expert Panel on the Care of Older Adults with Multimorbidity 2012).

Indicator type: Process quality

Quality dimension: Patient-centeredness, patient safety

Calculation of the indicator

Reference period: Before treatment decisions

Data collection: GP survey/documentation of discussion about potential benefits and harms of treatment option in medical records

Numerator: Number of patients who were informed about potential benefits and harms of treatment options before treatment decisions were made

Denominator: Number of patients aged 65 and over with three or more chronic conditions

Further inclusion and exclusion criteria: None

Indicator question(s): Did you discuss the potential benefits and risks of the (different) treatment options with your patient before the last decision in their treatment (e.g. performing a procedure, starting or stopping a long-term medication)?

□Yes □No □Not sure

Previous use and evidence

Underlying recommendation:

American Geriatrics Society Expert Panel (2012), p. 5: "Ensure that older adults with multimorbidity are adequately informed about the expected benefits and harms of different treatment options" *(strength of recommendation not available)*

Original indicator: Not available

Indicator: Information about medication

Specification

Description: Proportion (%) of patients aged 65 and over with three or more chronic conditions who were informed about their medicines (with regard to indication, effect and type of intake/use)

Target value: Maximum possible

Rationale: Uncertainty about medication intake can lead to non-adherence. Errors in intake or use are associated with risks for patients and may increase drug interactions.

Indicator type: Process quality

Quality dimension: Patient safety

Calculation of the indicator

Reference period: Upon new prescription

Data collection: Patient survey

Numerator: Number of patients who were informed about their medicines (with regard to indication, effect and type of intake/use).

Denominator: Number of patients aged 65 and over with three or more chronic conditions

Further inclusion and exclusion criteria: Patients without any prescribed medication are excluded

Indicator question(s): When you think about the last time your GP prescribed a new medication, what was it?

____

Has your GP explained to you how and why you should take the medication and how it works?

□ Yes □ No □ Not sure

Previous use and evidence

Underlying recommendation:

DEGAM Multimorbidity S3 Guideline (2017), p. 37: "In the case of pharmacological treatment, the medication actually used should be checked. (...) At the same time, any misunderstandings about the indication, effect and mode of intake or use of the medication should be clarified." *(moderate support for the recommendation)*

Original indicator: Not available

Indicator: Assessment of treatment burden

Specification

Description: Proportion (%) of patients aged 65 and over with three or more chronic conditions that had a discussion about their treatment burden

Target value: Maximum possible

Rationale: The presence of multiple chronic conditions implies a multitude of health-related tasks for many patients. The burden of treatments often interferes with symptom burden and functional limitations. If the amount of health-related tasks exceeds individual resources and capacities, the result can be overwhelm and non-adherence (German College of General Practitioners and Family Physicians 2017).

Notes: Contents of the conversation could be:

- the number and type of healthcare appointments a person has and where these take place
- the number and type of medicines a person is taking and how often,
- any harms from medicines
- non-pharmacological treatments such as diets, exercise programmes and psychological treatments
- any effects of treatment on their mental health or wellbeing

Indicator type: Process quality

Quality dimension: Patient-centeredness, patient safety

Calculation of the indicator

Reference period: 12 months

Data collection: GP survey/documentation of treatment burden in medical records, patient survey

Numerator: Number of patients that had a discussion about their treatment burden

Denominator: Number of patients aged 65 and over with three or more chronic conditions

Further inclusion and exclusion criteria: None

Indicator question(s): GP survey

Did you discuss with the patient the burden of their treatments?

(Note: The burden of treatment may include the following aspects:
- *the number and type of healthcare appointments a person has and where these take place*
- *the number and type of medicines a person is taking and how often,*
- *any harms from medicines*
- *non-pharmacological treatments such as diets, exercise programmes and psychological treatments*
- *any effects of treatment on their mental health or wellbeing)*

□ Yes, within the last 12 months □ Yes, more than 12 months ago
□ No □ Not sure

Patient survey

Patients with chronic conditions may also experience burden from medical treatment, e.g.: following diets and exercise programmes, taking medications and their side effects, seeing (different) doctors, etc.

Thinking about the last 12 months, has your GP discussed with you how you are coping with the burden of managing your chronic conditions?

□ Yes □ No □ Not sure

Previous use and evidence

Underlying recommendation:

NICE Multimorbidity: clinical assessment and management (2016), p. 237: "Establish treatment burden by talking to people about how treatments for their health problems affect their day-to-day life. Include in the discussion:
- the number and type of healthcare appointments a person has and where these take place
- the number and type of medicines a person is taking and how often,
- any harms from medicines
- non-pharmacological treatments such as diets, exercise programmes and psychological treatments
- any effects of treatment on their mental health or wellbeing" *(strong support for the recommendation)*

Original indicator: Not available

Indicator: Medication review

Specification

Description: Proportion (%) of patients aged 65 and over with three or more chronic conditions that received a review of their medication

Target value: Maximum possible

Rationale: The more medication is being taken, the higher the risk of drug interactions, adverse effects and non-adherence (German College of General Practitioners and Family Physicians 2017).

Notes: Long-term medication = At least one medication with a duration of use of at least 6 months

Indicator type: Process quality

Quality dimension: Patient safety, effectiveness of care

Calculation of the indicator

Reference period: 12 months

Data collection: GP survey/documentation of medication review in medical records, patient survey

Numerator: Number of patients who received a review of their medication

Denominator: Number of patients aged 65 and over with three or more chronic conditions receiving long-term medication

Further inclusion and exclusion criteria:

Only patients with long-term medication are considered (see notes)

Indicator question(s): GP survey

Did this patient receive a review of their medication?

□ Yes, within the last 12 months □ Yes, more than 12 months ago
□ No □ Not sure

Patient survey

In the last 12 months, has your GP checked with you what medicines you are taking, how well you tolerate them and how you manage their intake?

□ Yes □ No □ Not sure

□ Done by another provider: ____________________

Previous use and evidence

Underlying recommendation:

NICE Multimorbidity: clinical assessment and management (2016), p. 99: "Review medicines and other treatments taking into account evidence of likely benefits and harms for the individual patient and outcomes important to the person" *(strong support for the recommendation)*

Original indicator: *NICE Multimorbidity quality standard (2017), p. 15:* "Proportion of adults having a review of their medicines and other treatments for multimorbidity who discussed whether any can be stopped or changed"

Indicator: Monitoring of pain management

Specification

Description: Proportion (%) of patients aged 65 and over with three or more chronic conditions with chronic pain whose pain management was monitored and adjusted if necessary

Target value: Maximum possible

Rationale: Although pain is often underestimated by GPs, it is associated with negative effects on patients' quality of life, functioning and well-being. Often, pain in multimorbidity is influenced by several underlying factors, which can complicate effective pain management. However, continuous monitoring is essential in light of the potential impact of chronic pain (German College of General Practitioners and Family Physicians 2017).

Indicator type: Process quality

Quality dimension: Adequacy of care, effectiveness of care

Calculation of the indicator

Reference period: Three months

Data collection: GP survey/documentation of pain (treatment) monitoring in medical records

Numerator: Number of patients whose pain management was monitored and adjusted if necessary

Denominator: Number of patients aged 65 and over with three or more chronic conditions who suffer from chronic pain

Further inclusion and exclusion criteria: None

Indicator question(s): (If the patient suffers from chronic pain:) When did you last monitor the patient's pain management?

□ Within the last three months □ Within the last 12 months
□ More than 12 months ago □ Not sure
□ Does not apply

Previous use and evidence

Underlying recommendation:

NICE Multimorbidity: clinical assessment and management (2016), p. 237: "Be alert to the possibility of: chronic pain and the need to assess this and the adequacy of pain management" *(limited support for the recommendation)*

Original indicator: Not available

Indicator: Assigning responsibility for coordination of care

Specification

Description: Proportion (%) of patients aged 65 and over with three or more chronic conditions who have agreed upon which health care provider is responsible for the overall coordination of care

Target value: Maximum possible

Rationale: As central task in the care of patients with multimorbidity is to agree on the responsibility for coordination and to communicate this agreement to all service providers involved (National Institute for Health and Care Excellence 2016). Although GPs often take on this role, they only have limited time available to dedicate to this task (German College of General Practitioners and Family Physicians 2017).

Indicator type: Process quality

Quality dimension: Continuity of care, efficiency of care, adequacy of care

Calculation of the indicator

Reference period: Agreement once per patient and in the event of significant changes in the patient's life or care situation

Data collection: Patient survey

Numerator: Number of patients who have agreed upon which health care provider is responsible for the overall coordination of care

Denominator: Number of patients aged 65 and over with three or more chronic conditions

Further inclusion and exclusion criteria: None

Indicator question(s): Have you discussed with your GP who is responsible for coordinating your healthcare?

□ Yes □ No □ Not sure

Previous use and evidence

Underlying recommendation:

NICE Multimorbidity: clinical assessment and management (2016), p. 99: "Agree an individualised management plan with the person, including: (...) who is responsible for coordination of care" *(strong support for the recommendation).*

Original indicator: *NICE Multimorbidity quality standard (2017), p. 12:* "Adults with an individualised management plan for multimorbidity know who is responsible for coordinating their care"

Indicator: Training programmes addressing the management of pts with multimorbidity – physician staff

Specification

Description: At least one physician of the practice has participated in training programmes for multimorbidity

Target value: Criterion met for as many service providers/practices as possible

Rationale: Training relevant to multimorbidity care should help to consolidate and develop skills and knowledge relevant to the treatment of this patient group (Palmer et al. 2018).

Indicator type: Structural quality

Quality dimension: Evidence- and knowledge-based care

Calculation of the indicator

Reference period: Current status

Data collection: Practice survey/certificate of attendance

Calculation at the level of a supply unit

Criterion fulfilled/not fulfilled

Calculation at the level of a supply structure:

Numerator: Number of practices/care units for which at least one physician has participated in training programmes relevant to multimorbidity

Denominator: Number of all considered practices/care units of the care structure

Further inclusion and exclusion criteria:

Refers to practices/care units involved in the care of patients aged 65 and over with three or more chronic conditions

Indicator question(s): Has at least one physician in your practice taken part in training programmes relevant to multimorbidity?

□ Yes: ____
□ No
□ No information available

Previous use and evidence

Underlying recommendation:

JA-CHRODIS Multimorbidity care model (2016), p. 12: "Training of the members of the team (...) should focus on the following themes: comprehensive assessment, multimorbidity and its consequences, health outcomes, adverse effects and interactions of drugs, use of technologies, implementation of individualized care plans, goal setting, working effectively as a team, communication, training in the critical appraisal of knowledge and evidence-based knowledge, patient-centeredness, patient empowerment, and self-management" *(strength of recommendation not available)*

Original indicator: Not available

Indicator: Training programmes addressing the management of pts with multimorbidity – non-physician staff

Specification

Description: At least one member of the non-physician staff of a practice has participated in training programmes for multimorbidity

Target value: Criterion met for as many service providers/practices as possible

Rationale: Training relevant to multimorbidity care should help to consolidate and develop skills and knowledge relevant to the treatment of this patient group (Palmer et al. 2018).

Indicator type: Structural quality

Quality dimension: Evidence- and knowledge-based care

Calculation of the indicator

Reference period: Current status

Data collection: Practice survey/certificate of attendance

Calculation at the level of a supply unit:

Criterion fulfilled/not fulfilled

Calculation at the level of a supply structure:

Numerator: Number of practices/care units for which at least one member of the non-physician staff has participated in training programmes relevant to multimorbidity

Denominator: Number of all considered practices/care units of the care structure

Further inclusion and exclusion criteria:

Refers to practices/care units involved in the care of patients aged 65 and over with three or more chronic conditions

Indicator question(s): Has at least one member of your non-physician staff in your practice taken part in training programmes relevant to multimorbidity? (Please also note job title)

□ Yes: ____

□ No

□ No information available

Previous use and evidence

Underlying recommendation:

JA-CHRODIS Multimorbidity care model (2016), p. 12: "Training of the members of the team (...) should focus on the following themes: comprehensive assessment, multimorbidity and its consequences, health outcomes, adverse effects and interactions of drugs, use of technologies, implementation of individualized care plans, goal setting, working effectively as a team, communication, training in the critical appraisal of knowledge and evidence-based knowledge, patient-centeredness, patient empowerment, and self-management" *(strength of recommendation not available)*

Original indicator: Not available

References

American Geriatrics Society Expert Panel on the Care of Older Adults with Multimorbidity (2012): Guiding Principles for the Care of Older Adults with Multimorbidity: An Approach for Clinicians. In *Journal of the American Geriatrics Society* 60 (10), E1-E25. DOI: 10.1111/j.1532-5415.2012.04188.x.

Cleeland, Charles S. (2007): Symptom burden: multiple symptoms and their impact as patient-reported outcomes. In *Journal of the National Cancer Institute. Monographs* (37), pp. 16–21. DOI: 10.1093/jncimonographs/lgm005.

German College of General Practitioners and Family Physicians (DEGAM) (2017): Multimorbidität. S3-Leitlinie. Available online at https://www.awmf.org/leitlinien/detail/ll/053-047.html, checked on 12/9/2020.

German College of General Practitioners and Family Physicians (DEGAM) (2021): Hausärztliche Leitlinie Multimedikation. Available online at https://www.awmf.org/leitlinien/detail/ll/053-043.html, checked on 6/23/2022.

Muth, Christiane; van den Akker, Marjan; Blom, Jeanet W.; Mallen, Christian D.; Rochon, Justine; Schellevis, François G. et al. (2014): The Ariadne principles: how to handle multimorbidity in primary care consultations. In *BMC Medicine* 12 (1), p. 223. DOI: 10.1186/s12916-014-0223-1.

National Institute for Health and Care Excellence (NICE) (2016): Multimorbidity: clinical assessment and management. Available online at https://www.nice.org.uk/guidance/ng56, checked on 12/9/2020.

National Institute for Health and Care Excellence (NICE) (2017): Multimorbidity quality standard. Available online at https://www.nice.org.uk/guidance/qs153, checked on 12/7/2020.

Palmer, Katie; Marengoni, Alessandra; Forjaz, Maria João; Jureviciene, Elena; Laatikainen, Tiina; Mammarella, Federica et al. (2018): Multimorbidity care model: Recommendations from the consensus meeting of the Joint Action on Chronic Diseases and Promoting Healthy Ageing across the Life Cycle (JA-CHRODIS). In *Health Policy* 122 (1), pp. 4–11. DOI: 10.1016/j.healthpol.2017.09.006.

Palmer, Katie; Marengoni, Alessandra; Jureviciene, Elena; Laatikainen, Tiina; Mammarella, Federica; Muth, Christiane et al. (2016): Multimorbidity care model. Recommendations from the consensus meeting of the Joint Action on Chronic. Joint Action CHRODIS. Available online at http://chrodis.eu/our-work/06-multimorbidity/wp06-activities/multimorbiditycaremodel/, checked on 6/23/2022.

Working Group on Health Outcomes for Older Persons with Multiple Chronic Conditions (2012): Universal health outcome measures for older persons with multiple chronic conditions. In *Journal of the American Geriatrics Society* 60 (12), pp. 2333–2341. DOI: 10.1111/j.1532-5415.2012.04240.x.

World Health Organization (2003): Adherence to long-term therapies : evidence for action / [edited by Eduardo Sabaté]. Geneva: World Health Organization. Available online at https://apps.who.int/iris/handle/10665/42682.
