# Supplementary material for: Validation of patient- and GP-reported core sets of quality indicators for older adults with multimorbidity in primary care: results of the cross-sectional observational MULTIqual validation study
Source: BMC Med. 2023 Apr 17;21:148. doi: 10.1186/s12916-023-02856-0 (PMC10111827; doi:10.1186/s12916-023-02856-0)
Supplement: Supplementary file 2 — Additional file 2. Questionnaires. [file 12916_2023_2856_MOESM2_ESM.pdf]

VALIDATION OF PATIENT- AND GP-REPORTED CORE SETS OF QUALITY INDICATORS FOR OLDER ADULTS WITH MULTIMORBIDITY IN PRIMARY CARE: RESULTS OF THE CROSS-SECTIONAL OBSERVATIONAL MULTIQUAL VALIDATION STUDY

Additional file 2: Questionnaires

QUESTIONNAIRE FOR GP-REPORTED CORE SETS OF QUALITY INDICATORS FOR OLDER ADULTS WITH MULTIMORBIDITY IN PRIMARY CARE (ENGLISH VERSION)

1. Proactive pain assessment

When was the patient last asked about the presence of pain?

□ Within the last three months □ Within the last 12 months
□ More than 12 months ago □ Not sure

2. Involving partner, family and caregivers

Has it been discussed with the patient in general whether and to what extent partners, family and caregivers should be involved in important decisions regarding their care?

□ Yes □ No □ Not sure

What was the last significant change in the patient's health status? *(Note: Several may apply)*

□Deterioration of health status □ Improvement in health status
□Hospitalisation □New diagnosis
□ Other: _______________________

On this occasion, has it been discussed again whether and to what extent partners, family and caregivers should be involved in important decisions about their care?

□ Yes □ No □ Not sure

3. Monitoring adherence to treatment

Did you have a discussion with the patient about their adherence to treatment?

□ Yes, within the last 12 months □ Yes, more than 12 months ago
□ No □ Not sure

4. Quality of life assessment

Did you have a discussion with the patient about their perceived quality of life?

□ Yes, within the last 12 months □ Yes, more than 12 months ago
□ No □ Not sure

5. Eliciting patient preferences

Did you have a discussion with the patient about their preferences (values, goals and priorities) regarding their health care?

(Note: Preferences may include, e.g., maintaining employment, participating in social activities/family life, preventing adverse events (e.g., stroke), minimising adverse effects of medications, reducing burden of treatments, prolonging life.)

□ Yes, within the last three months □ Yes, within the last 12 months
□ Yes, more than 12 months ago □ No
□ Not sure

6. Mutual agreement on treatment goals

Did you agree on treatment goals together with the patient?

□ Yes, within the last three months □ Yes, within the last 12 months
□ Yes, more than 12 months ago □ No
□ Not sure

7. Information about potential benefits and harms of treatment options

Did you discuss the potential benefits and risks of the (different) treatment options with your patient before the last decision in their treatment (e.g., performing a procedure, starting or stopping a long-term medication)?

□Yes □No □Not sure

8. Assessment of treatment burden

Did you discuss with the patient the burden of their treatments?

(Note: The burden of treatment may include the following aspects:

- *the number and type of healthcare appointments a person has and where these take place*
- *the number and type of medicines a person is taking and how often,*
- *any harms from medicines*
- *non-pharmacological treatments such as diets, exercise programmes and psychological treatments*
- *any effects of treatment on their mental health or wellbeing)*

□ Yes, within the last 12 months □ Yes, more than 12 months ago
□ No □ Not sure

9. Monitoring of pain management

Does the patient suffer from chronic pain?

□Yes □No □Not sure

If yes: When did you last monitor the patient's pain management?

□ Within the last three months □ Within the last 12 months
□ More than 12 months ago □ Not sure
□ Does not apply

10. Medication review

Did this patient receive a review of their medication?

□ Yes, within the last 12 months □ Yes, more than 12 months ago
□ No □ Not sure

11. Training of physician staff addressing multimorbidity

Has at least one physician in your practice taken part in training programmes relevant to multimorbidity?

□ Yes: ____
□ No
□ No information available

12. Training of non-physician staff addressing multimorbidity

Has at least one member of your non-physician staff in your practice taken part in training programmes relevant to multimorbidity? (Please also note job title)

□ Yes: ____
□ No
□ No information available

QUESTIONNAIRE FOR PATIENT-REPORTED CORE SETS OF QUALITY INDICATORS FOR OLDER ADULTS WITH MULTIMORBIDITY IN PRIMARY CARE (ENGLISH VERSION)

1. **Proactive pain assessment**

Thinking about the last three months, has your GP asked you specifically about the presence of pain?

□ Yes □ No □ Not sure

2. **Eliciting patient preferences**

Thinking about the last three months, has your GP asked you about your wishes, health goals and what is important to you in your treatment?
(Note: Examples may include participation in social activities, family life, prevention of negative events such as strokes or falls, minimisation of medication side effects, pain reduction, etc.)

□ Yes □ No □ Not sure

3. **Mutual agreement on treatment goals**

Thinking about the last three months, have you discussed your treatment goals with your GP?

□ Yes □ No □ Not sure

4. **Information about medication**

When you think about the last time your GP prescribed a new medication, what was it?

____

Has your GP explained to you how and why you should take the medication and how it works?

□ Yes □ No □ Not sure

5. **Assessment of treatment burden**

Patients with chronic conditions may also experience burden from medical treatment, e.g.: following diets and exercise programmes, taking medications and their side effects, seeing (different) doctors, etc.

Thinking about the last 12 months, has your GP discussed with you how you are coping with the burden of managing your chronic conditions?

□ Yes □ No □ Not sure

6. Medication review

In the last 12 months, has your GP checked with you what medicines you are taking, how well you tolerate them and how you manage their intake?

□ Yes □ No □ Not sure

□ Done by another provider: ____________________

7. Assigning responsibility for coordination of care

Have you discussed with your GP who is responsible for coordinating your healthcare?

□ Yes □ No □ Not sure

QUALITÄTSINDIKATOREN FÜR MULTIMORBIDITÄT

FRAGEBOGEN FÜR HAUSÄRZTINNEN/HAUSÄRZTE

(DEUTSCHE VERSION)

1. Gezieltes Erfassen von Schmerzen

Wann wurde der Patient/die Patientin zuletzt auf das Vorliegen von Schmerzen befragt?

□ Innerhalb der letzten drei Monate
□ Innerhalb der letzten 12 Monate
□ Vor mehr als 12 Monaten
□ Nicht sicher

2. Einbeziehen von Bezugspersonen

Wurde mit der Patientin/dem Patienten grundsätzlich besprochen, ob und inwieweit Bezugspersonen in wichtige Versorgungsentscheidungen einbezogen werden sollen?

□ Ja □ Nein □ Weiß nicht

Was war die letzte einschneidende Veränderung im gesundheitlichen Zustand des Patienten/der Patientin? *(Hinweis: Es können mehrere zutreffen)*

□Verschlechterung des Gesundheitszustands
□Verbesserung des Gesundheitszustands
□Krankenhausaufenthalt
□Neudiagnose
□ Sonstiges: ________________________

Wurde mit dem Patienten/der Patientin aus diesem Anlass (erneut) besprochen, ob und inwieweit Bezugspersonen in wichtige Versorgungsentscheidungen einbezogen werden sollen?

□ Ja □ Nein □ Weiß nicht

3. Überprüfung der Therapieadhärenz

Haben Sie im Gespräch die Therapie-Adhärenz Ihrer Patientin/Ihres Patienten thematisiert? *(Hinweis: Unter Adhärenz verstehen wir das Ausmaß, in dem das Verhalten eines Patienten mit zuvor zwischen Arzt oder Therapeuten gemeinsam festgelegten Behandlungszielen und -wegen übereinstimmt.)*

□ Ja, innerhalb der letzten 12 Monate
□ Ja, vor mehr als 12 Monaten
□ Nein
□ Weiß nicht

4. Einschätzen der subjektiven Lebensqualität

Haben Sie mit dem Patienten/der Patientin über seine/ihre subjektive Lebensqualität gesprochen?

□ Ja, innerhalb der letzten 12 Monate
□ Ja, vor mehr als 12 Monaten
□ Nein
□ Weiß nicht

5. Ermitteln von Patientenpräferenzen

Haben Sie bei der Patientin/dem Patienten Präferenzen (Werte, Ziele und Prioritäten) in Bezug auf die gesundheitliche Versorgung erfragt?
(Hinweis: Präferenzen können beispielsweise sein: Erhalt der Arbeitstätigkeit, Teilnahme an sozialen Aktivitäten/ Familienleben, Verhinderung von spezifischen Ereignissen (z.B. Schlaganfall), Minimierung von Medikamentennebenwirkungen, Verringerung der Belastung durch Behandlungen, Lebensverlängerung.)

□ Ja, innerhalb der letzten drei Monate
□ Ja, innerhalb der letzten 12 Monate
□ Ja, vor mehr als 12 Monaten
□ Nein
□ Weiß nicht

6. Gemeinsame Vereinbarung von Behandlungszielen

Haben Sie gemeinsam mit der Patientin/dem Patienten Behandlungsziele vereinbart?

□ Ja, innerhalb der letzten drei Monate
□ Ja, innerhalb der letzten 12 Monate
□ Ja, vor mehr als 12 Monaten
□ Nein
□ Weiß nicht

7. Informationen über potentiellen Nutzen und Schaden von Therapieoptionen

Haben Sie mit Ihrem Patienten/Ihrer Patientin vor der letzten Behandlungsentscheidung (z. B. Durchführung eines Eingriffs, Beginn oder Absetzen eines Langzeitmedikaments) die potenziellen Vorteile und Risiken der (verschiedenen) Behandlungsoptionen besprochen?

□ Ja □ Nein □ Weiß nicht

8. Erhebung der Belastung durch die Behandlungen

Haben Sie mit der Patientin/dem Patienten darüber gesprochen, inwiefern es durch die Behandlungen auch zu Belastungen kommt?
(*Hinweis: Die Belastung durch die Behandlungen kann folgende Faktoren beinhalten:*

- *Anzahl und Art der Behandlungstermine einer Person und wo diese stattfinden*
- *Anzahl und Art der Arzneimittel, die eine Person einnimmt, und wie oft*
- *unerwünschte Wirkungen durch Arzneimittel*
- *nichtpharmakologische Behandlungen wie Diäten, Bewegungsprogramme und psychologische Behandlungen*
- *Auswirkungen der Behandlung auf die psychische Gesundheit oder das Wohlbefinden)*

□ Ja, innerhalb der letzten 12 Monate
□ Ja, vor mehr als 12 Monaten
□ Nein
□ Weiß nicht

9. Überprüfen des Schmerzmanagements

Leidet der Patient/die Patientin an chronischen Schmerzen?

□ Ja □ Nein □ Weiß nicht

Wenn ja: Wann haben Sie die Schmerzbehandlung des Patienten/der Patientin zuletzt überwacht?

□ Innerhalb der letzten drei Monate
□ Innerhalb der letzten 12 Monate
□ Vor mehr als 12 Monaten
□ Nicht sicher
□ Nicht zutreffend

10. Medikamentenreview

Wurde bei dieser Patientin/diesem Patienten ein strukturiertes Medikamentenreview vorgenommen?
(Hinweis: Umfasst die strukturierte Bewertung der eingenommenen Medikation nach der Indikation, möglichen Kontraindikationen und Interaktionen, Dosierung und Angemessenheit der Therapie.)

□ Ja, innerhalb der letzten 12 Monate □ Ja, vor mehr als 12 Monaten
□ Nein □ Weiß nicht

11. Fortbildungsmaßnahmen mit Relevanz für Multimorbidität –ärztliches Personal

Hat mindestens ein/e Mitarbeiter/in aus dem Personal Ihrer Praxis an Fortbildungsmaßnahmen mit Relevanz für Multimorbidität teilgenommen?
(Hinweis: Fortbildungsmaßnahmen mit Relevanz für Multimorbidität beinhalten z.B. umfassendes Assessment, Multimorbidität und ihre Auswirkungen, Krankheitsfolgen, unerwünschte Arzneimittelwirkungen und Medikamenteninteraktionen, Nutzung von technischen Unterstützungssystemen, Anwendung individuell zugeschnittener Behandlungspläne, Zielvereinbarung, effektive Zusammenarbeit im Team, Kommunikationstraining, Evidenzbasierte Medizin, Patientenzentrierung, Patienten-Empowerment, Selbstmanagement)

□ Ja: ____
□ Nein
□ Keine Informationen verfügbar

12. Fortbildungsmaßnahmen mit Relevanz für Multimorbidität – nichtärztliches Personal

Hat mindestens ein Mitglied des nichtärztlichen Personals in Ihrer Praxis an Fortbildungsmaßnahmen zum Thema Multimorbidität teilgenommen?

□ Ja: ____
□ Nein
□ Keine Informationen verfügbar

QUALITÄTSINDIKATOREN FÜR MULTIMORBIDITÄT

FRAGEBOGEN FÜR PATIENTINNEN/PATIENTEN

(DEUTSCHE VERSION)

1. **Gezieltes Erfassen von Schmerzen**

Hat Ihr Hausarzt/Ihre Hausärztin Sie in den letzten drei Monaten gezielt danach gefragt, ob bei Ihnen Schmerzen vorliegen?

□ Ja □ Nein □ Nicht sicher

2. **Ermitteln von Patientenpräferenzen**

Wenn Sie an die letzten drei Monate denken, hat Ihr Hausarzt/Ihre Hausärztin Sie danach gefragt, welche Wünsche und gesundheitlichen Ziele Sie haben und was Ihnen in der Behandlung wichtig ist? *(Hinweis: Beispiele können sein: Teilnahme an sozialen Aktivitäten, Familienleben, Verhinderung von negativen Ereignissen wie Schlaganfällen oder Stürzen, Minimierung von Medikamentennebenwirkungen, Schmerzreduktion.)*

□ Ja □ Nein □ Weiß nicht

3. **Gemeinsame Vereinbarung von Behandlungszielen**

Wenn Sie an die letzten drei Monate denken, haben Sie gemeinsam mit Ihrem Hausarzt/Ihrer Hausärztin Behandlungsziele vereinbart?

□ Ja □ Nein □ Weiß nicht

4. **Information über Medikation**

Wenn Sie an das letzte Mal denken, als Ihr Hausarzt/Ihre Hausärztin ein neues Medikament verschrieben hat, was war das?

____

Hat Ihr Hausarzt/Ihre Hausärztin Ihnen erklärt, wie und warum Sie das Medikament einnehmen sollten und wie es wirkt?

□ Ja □ Nein □ Weiß nicht

5. Erhebung der Belastung durch die Behandlungen

Patienten mit chronischen Erkrankungen können durch die medizinische Behandlung auch Belastungen erfahren, z.B. durch:

- das Einhalten von Diäten und Bewegungsprogrammen
- die Einnahme von Medikamenten und deren Nebenwirkungen
- Termine bei (verschiedenen) Ärzten etc.

Wenn Sie an die letzten 12 Monate denken, hat Ihre Hausärztin/Ihr Hausarzt mit Ihnen darüber gesprochen, ob Sie mit der Belastung durch die Behandlung Ihrer chronischen Erkrankungen zurechtkommen?

□ Ja □ Nein □ Weiß nicht

6. Strukturiertes Medikamentenreview

Hat Ihr Hausarzt/Ihre Hausärztin in den letzten zwölf Monaten gemeinsam mit Ihnen überprüft, welche Medikamente Sie einnehmen, ob Sie die Medikamente gut vertragen und mit der Anwendung zurechtkommen?

□ Ja □ Nein □ Weiß nicht

□ Hat eine andere Fachperson gemacht: ___________

7. Festlegung der Verantwortlichkeit für die Koordination

Haben Sie mit Ihrem Hausarzt/Ihrer Hausärztin besprochen, wer dafür verantwortlich ist, Ihre medizinische Versorgung zu koordinieren?

□ Ja □ Nein □ Weiß nicht
